# Good Statistical Practice—development of tailored Good Clinical Practice training for statisticians

**DOI:** 10.1186/s13063-024-07940-1

**Published:** 2024-02-10

**Authors:** Deborah D. Stocken, Helen Mossop, Emma Armstrong, Steff Lewis, Susan J. Dutton, Claire Peckitt, Carrol Gamble, Julia Brown

**Affiliations:** 1 Leeds Institute of Clinical Trials Research, Faculty of Medicine and Health, University of Leeds, Leeds, UK; 2 Biostatistics Research Group, Institute of Health and Society, Newcastle University, Newcastle Upon Tyne, UK; 3 Edinburgh Clinical Trials Unit, Usher Institute of Population Health Sciences and Informatics, University of Edinburgh, Edinburgh, UK; 4 Oxford Clinical Trials Research Unit, Nuffield Department of Orthopaedics, Rheumatology and Musculoskeletal Sciences, University of Oxford, Oxford, UK; 5 Royal Marsden and Institute for Cancer Research Clinical Trials Units, The Royal Marsden NHS Foundation Trust, London, UK; 6 Liverpool Clinical Trials Clinical Trials Research Centre, Department of Biostatistics, University of Liverpool, Liverpool, UK

**Keywords:** Good clinical practice, Good statistical practice, Clinical research

## Abstract

**Background:**

Statisticians are fundamental in ensuring clinical research, including clinical trials, are conducted with quality, transparency, reproducibility and integrity. Good Clinical Practice (GCP) is an international quality standard for the conduct of clinical trials research. Statisticians are required to undertake training on GCP but existing training is generic and, crucially, does not cover statistical activities. This results in statisticians undertaking training mostly unrelated to their role and variation in awareness and implementation of relevant regulatory requirements with regards to statistical conduct. The need for role-relevant training is recognised by the UK NHS Health Research Authority and the Medicines and Healthcare products Regulatory Agency (MHRA).

**Methods:**

The Good Statistical Practice (GCP for Statisticians) project was instigated by the UK Clinical Research Collaboration (UKCRC) Registered Clinical Trials Unit (CTU) Statisticians Operational Group and funded by the National Institute for Health and Care Research (NIHR), to develop materials to enable role-specific GCP training tailored to statisticians. Review of current GCP training was undertaken by survey. Development of training materials were based on MHRA GCP. Critical review and piloting was conducted with UKCRC CTU and NIHR researchers with comment from MHRA. Final review was conducted through the UKCRC CTU Statistics group.

**Results:**

The survey confirmed the need and desire for the development of dedicated GCP training for statisticians. An accessible, comprehensive, piloted training package was developed tailored to statisticians working in clinical research, particularly the clinical trials arena. The training materials cover legislation and guidance for best practice across all clinical trial processes with statistical involvement, including exercises and real-life scenarios to bridge the gap between theory and practice. Comprehensive feedback was incorporated. The training materials are freely available for national and international adoption.

**Conclusion:**

All research staff should have training in GCP yet the training undertaken by most academic statisticians does not cover activities related to their role. The Good Statistical Practice (GCP for Statisticians) project has developed and extensively piloted new, role-specific, comprehensive, accessible GCP training tailored to statisticians working in clinical research, particularly the clinical trials arena. This role-specific training will encourage best practice, leading to transparent and reproducible statistical activity, as required by regulatory authorities and funders.

## Background

Statisticians play a crucial role in clinical trials research across all stages of design, delivery, analysis and reporting. The key role of statisticians is recognised by regulatory agencies [1, 2] and funding bodies.

Good Clinical Practice (GCP) is an international quality standard [1] which applies throughout all stages and across all disciplines involved in clinical trials. UK regulations [3] set ethical and scientific standards stating all clinical trials of investigational medicinal products (CTIMPs) are required by law to be conducted in accordance with the principles of GCP. All research staff, including research statisticians, should be trained in GCP and an awareness of GCP requirements in relation to their role [2, 4, 5]. Whilst there is no legal requirement for non-CTIMPS, UK policy [6] sets out principles of good practice [4] for all health research to ensure the rights, safety and wellbeing of research participants are protected and that the research is reliable. GCP awareness, compliance and training is necessary for all research statisticians yet current GCP training is aimed at site delivery staff in generating data so results are credible, reproducible and reliable.

Despite the recognised key role of the statistician, GCP training undertaken by most academic statisticians is generic and aimed at site delivery staff. Crucially, training does not cover activities related to their role. There is no GCP training specific to statistical principles described by European Medicines Agency (EMA) International Council for Harmonisation of Technical Requirements for Pharmaceuticals for Human Use (ICH) [1] E9 scientific guideline for statistical principles for clinical trials, Medicines and Healthcare products Regulatory Agency (MHRA) GCP [2] or Medical Research Council (MRC) GCP [7]. Despite receiving GCP certification, there is a lack of clarity regarding the practical interpretation and translation of the principles of GCP into practice. Research statisticians need dedicated, role-specific GCP training and guidance to effectively translate GCP principles into practice to deliver statistical activities with transparency, integrity and consistently across a trial compliant with GCP recommendations. The need for role-specific training is supported by the Health Research Authority (HRA) [5] and the MHRA [2] who acknowledge that GCP training does not need to follow a generic syllabus or format and can be tailored to individuals’ roles and responsibilities.

The UK Clinical Research Collaboration (UKCRC) Registered Clinical Trials Unit (CTU) Statisticians Operational Group raised GCP training for statisticians as a high-priority training need. This paper describes the development of accessible, comprehensive, piloted Good Statistical Practice (GSP) training materials tailored to statisticians working in clinical research, particularly the clinical trials arena. The training equips statisticians with relevant regulatory knowledge to strengthen GCP interpretation and implementation in relation to statistician responsibilities. This training is directly relevant to all statisticians working in the medical arena and is freely available for national and international adoption.

## Methods

### Review of current GCP training

**Fig. 1:**
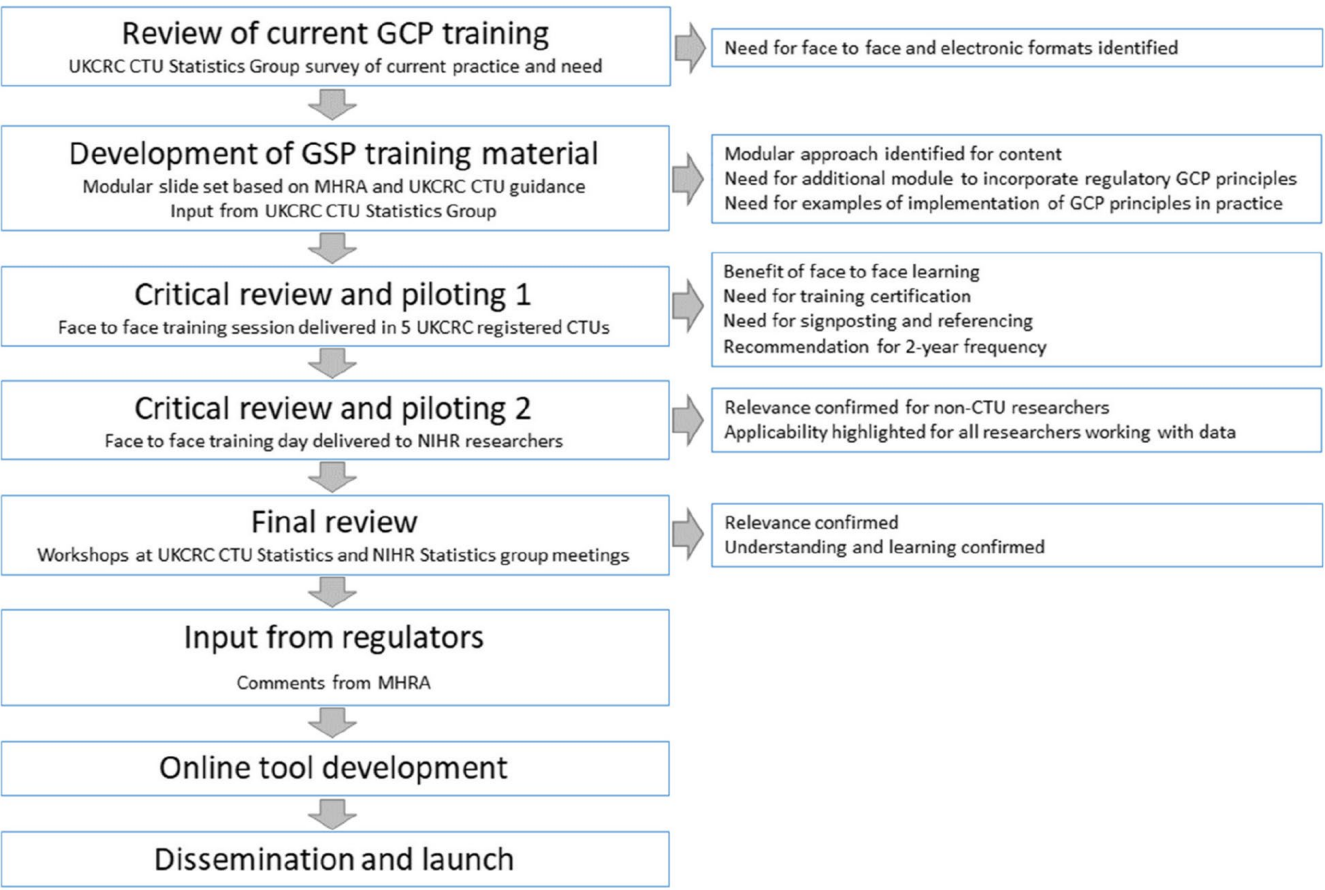
GSP development plan

Statistical activities and processes are not covered explicitly by existing GCP training accessible to those working in the UK National Health Service, universities and other publicly funded organisations involved in conducting clinical research. A survey of senior statisticians across the UKCRC CTU network, conducted at the bi-annual meetings, identified current training practices, perceived need for role-specific training and elicited opinion of training preferences. A successful funding application was based on a development plan as summarised in Fig. 1.

### Design and development of GSP training material

The training material was developed, by experienced clinical trials statisticians, as a version-controlled series of slides, according to a modular, accompanied with detailed notes and references. The focus of the training material was initially based on feedback from the UKCRC CTU survey and recognised needs of the experienced co-applicant team. The content was developed utilising the MHRA guidance [2] which details legislative requirements and processes compliant with the principles of GCP specific to the conduct of CTIMPs in the UK. Other relevant good statistical practice guidance documents developed by the UKCRC CTU network [8–12] and relevant published guidance [13–17] were included.

In the first instance, areas directly applicable to the roles and responsibilities of a clinical trial statistician were identified, followed by areas where there would be an expectation for statistical involvement, oversight or awareness, either because this was stated explicitly by MHRA or as identified through UKCRC CTU Statistics group activities. Development activities were conducted over a number of face-to-face UKCRC CTU Statistics group bi-annual meetings attended by 1 or 2 senior statisticians from each of the 45 UKCRC CTUs registered at that time. Development activities focussed on the practical implementation of specific GCP requirements based on group discussion of real-life experiences and scenarios from experienced statisticians influencing the examples, scenarios and suggestions used in further iterations of the material prior to piloting.

Feedback included a desire for training to be stand-alone, as opposed to an adjunct to existing GCP training. This resulted in an additional generic module on core GCP principles to be developed.

From the outset, it was acknowledged the delivery of the training material would need to be flexible, including group face to face as well as individual e-learning formats, to address differing statistical environments.

### Critical review and piloting

Following development, the training material was piloted in five UKCRC Registered CTUs. A senior statistician within each CTU delivered draft versions of the slide content as face-to-face training to their statistics team, as small group activity, with discussion regarding the understanding, relevance and practical implementation of content against CTU-specific standard operation processes. The senior statistical CTU lead provided collective, informal, detailed feedback to identify gaps or ambiguities or limits in implementation in practice.

**Fig. 2:**
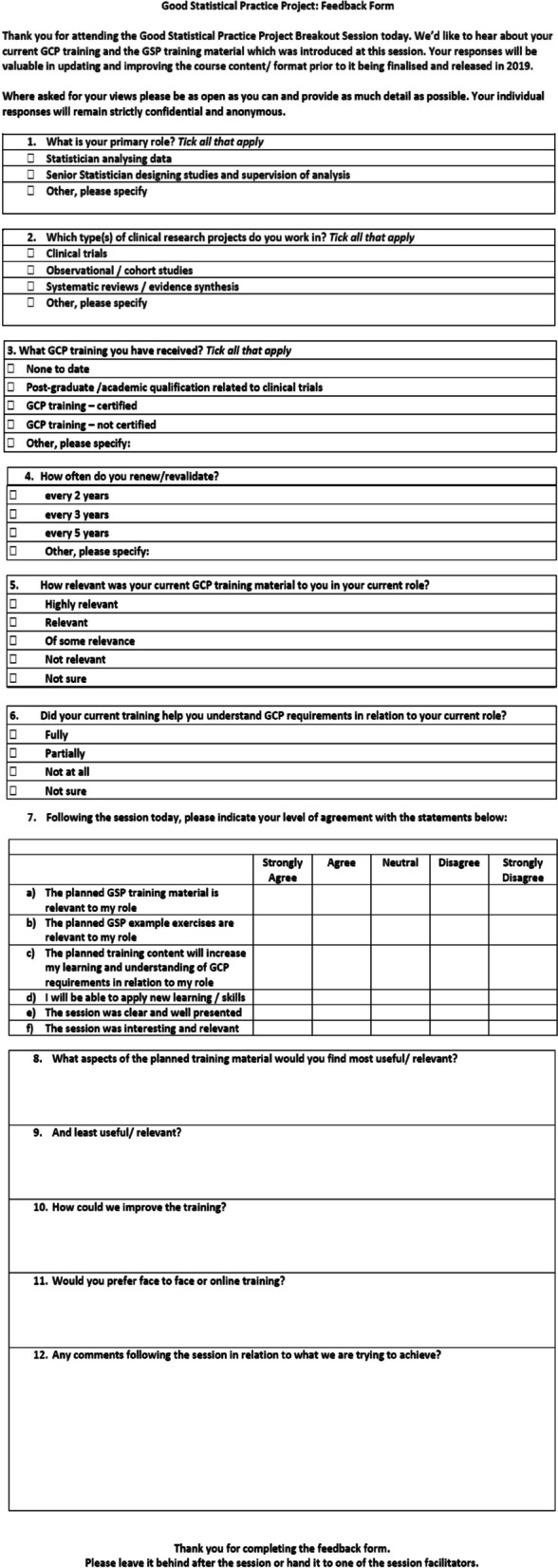
Structured feedback form

Further pilot activity was conducted with National Institute for Health and Care Research (NIHR) statisticians to ensure the training remained relevant to statisticians conducting research outside of the UKCRC Registered CTU network. A day long training course, based on updated draft versions of the slide content and accompanied detailed notes, was delivered by members of the development team to statisticians working on non-clinical trial NIHR research. Training material was interjected with small group exercises and structured, anonymous feedback (Fig. 2) was collated.

The format and content of the training was updated after both UKCRC CTU and NIHR pilot work and presented at break-out workshop sessions of the UKCRC CTU Statistics Groups and the NIHR Statistics Group meetings. Structured, anonymous feedback was collated into a final draft which was circulated to the MHRA for comment.

## Results

### Review of current GCP training

An initial scoping exercise with UKCRC CTU Statisticians highlighted that, although interesting and research related, the GCP training statisticians received was felt to be unrelated to their statistical role. At a bi-annual meeting attended by at least 1 senior statistician from each of the 45 UKCRC CTUs registered at that time, all but one attendee felt there was a need for more role specific training. A follow-on survey of 45 statisticians representing the 45 UKCRC CTUs, completed at the next bi-annual meeting, confirmed the need and clear desire for the development of a dedicated GCP training for statisticians (Table 1). The majority of responders, 34 (76%), were senior statisticians responsible for designing trials and supervising analyses; 11 (24%) were statisticians responsible for analysing trial data; 22 (49%) had worked in clinical trials for more than 10 years; 10 (22%) 5–10 years and 39 (87%) worked on CTIMP trials.

**Table 1:** Survey responses

	*N* = 45 CTU representatives	%
Primary role		
Senior statistician	34	76%
Statistician	11	24%
Years worked in clinical trials		
< 1 year	4	9%
1–5 years	9	20%
5–10 years	10	22%
> 10 years	22	49%
Predominantly working in clinical trials in:		
CTIMP	39	87%
Surgical	23	51%
Medical device	20	44%
GCP training received (yes)	44	98%
Type(s) of GCP training received^a^		
NIHR (face-to-face or online)	26	57%
In-house	19	43%
Institute of Clinical Research (ICR)	5	11%
Relevance of GCP training to role		
Highly relevant	5	11%
Relevant	10	23%
Some relevance	25	57%
Not relevant	3	7%
Not sure	1	2%
How much did training help you understand GCP requirements in relation to your role		
Fully	7	16%
Partially	31	70%
Not at all	5	11%
Not sure	1	2%
Do you think statisticians need a dedicated GCP training course		
Yes	30	68%
No	4	9%
Not sure	10	23%
Preferred form of GCP training^a^		
Online only	25	56%
Face-to-face only	20	44%
Reading based	6	13%
No preference	4	9%
Preferred audience(s) for GCP training^a^		
Role specific (statistician)	31	70%
Multi-professional—restricted to CTU teams	13	30%
Multi-professional—not restricted	4	9%
Not answered	1	

^a^Not mutually exclusive

One responder had not received any GCP training, but of those that had, 21 (48%) reported they had received certified GCP training and 19 (43%) received in-house training. Not exclusively, 15 (34%) attended NIHR-certified face-to-face GCP training, 11 (25%) NIHR-certified online GCP training and 5 (11%) attended Institute of Clinical Research (ICR)-certified online training. NIHR GCP training is designed for individuals involved in the conduct of studies at research sites, and NIHR acknowledge their training will not prepare those who have responsibility for other elements of a study.

Crucially, of 44 recipients, only five (11%) considered the GCP training they had received was highly relevant to their role, and only seven (16%) thought it helped them understand GCP requirements related to their role. The development of a dedicated GCP training course for statisticians was supported by 30 (68%) statisticians; only 4 (9%) thought there was no need for a dedicated GCP training course.

Respondents were asked to choose their preferred form(s) of GCP training with 25 (56%) stating online, 20 (44%) face-to-face and 6 (13%) reading/workbook based; 4 (9%) reported no preference. Collective team training within statistical teams were preferred by 31 (69%); 13 (29%) preferred multi-professional CTU restricted teams; 4 (9%) multi-professional and un-restricted.

### Design and development of GSP training material

A comprehensive set of training material has been developed to provide training for the translation of GCP into practice for statisticians involved in the conduct and analysis of clinical research in the UK. The training material has been developed by an experienced team of statisticians with knowledge of UK regulators and funders and in consultation with NIHR Learn and MHRA. It provides a high-level overview of GCP requirements and recommendations for best statistical practice.

**Fig. 3:**
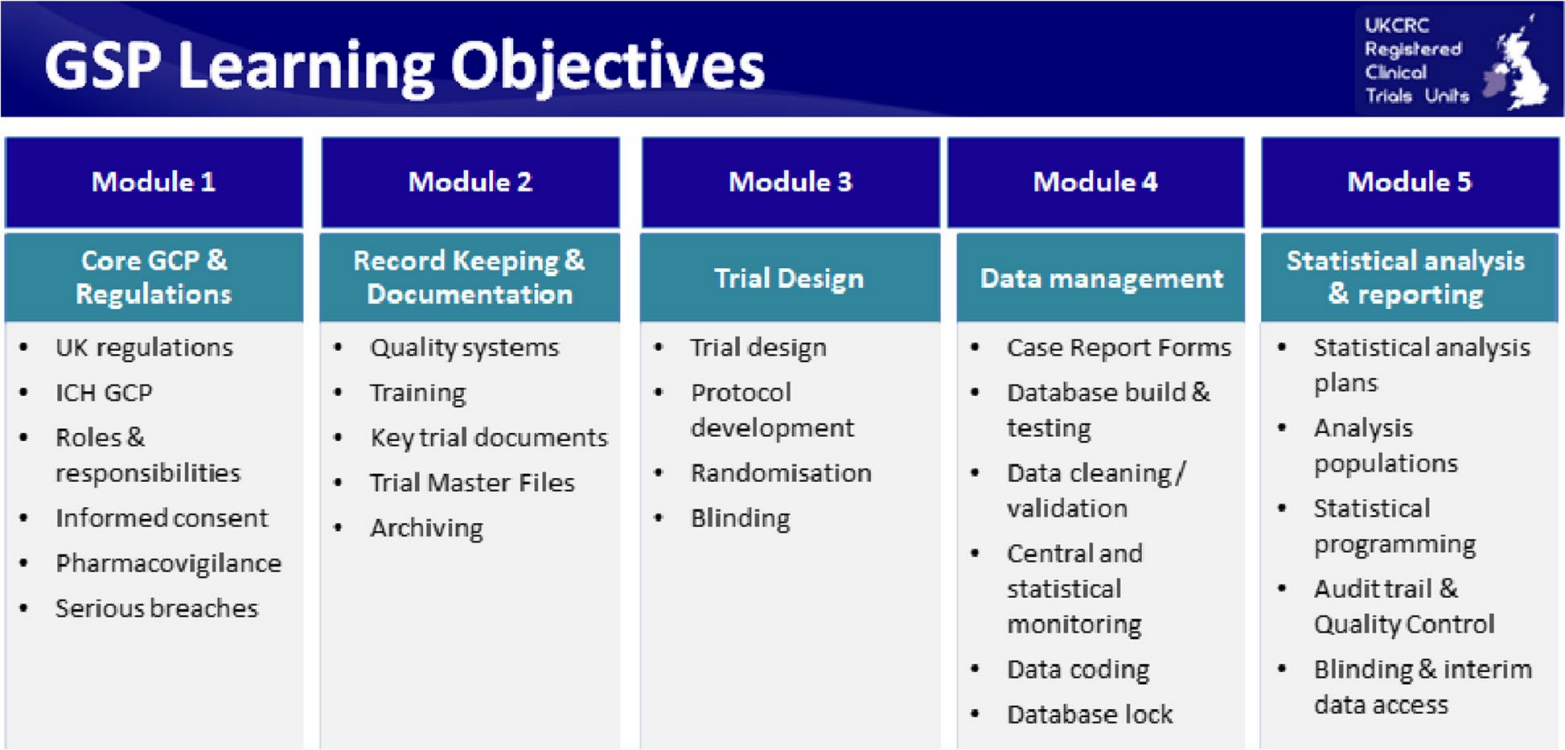
Modular training structure

The training material was developed through a version-controlled process to a final set of 114 slides, separated by learning objectives into five subject-specific modules, accompanied with detailed notes, specifically: (i) Module 1: Core GCP and Regulations; (ii) Module 2: Record Keeping and Documentation; (iii) Module 3: Trial Design; (iv) Module 4: Data Management and (v) Module 5: Statistical Analysis and Reporting. The five modules (Fig. 3, Table 2) focus on GCP requirements or recommendations directly related to statistical activities, or activities which would usually require some statistical involvement, as well as additional topics applicable to staff working in research more generally, but of which statistical staff should have an awareness.

**Table 2:** Training topics and modules

**Areas of direct relevance to statisticians**
□ Requirement for a statistical analysis plan (SAP) and recommendations around timing of sign-off (Module 5)
□ Documentation of protocol non-compliances and exclusions from per-protocol populations (Module 5)
□ Processes and documentation to be in place for formal interim analyses (Module 4)
□ Recommendations for blinding and interim data access, e.g. for data monitoring committee reports (Module 5)
□ Security of datasets and analysis files (Module 5)
□ Recommendations for statistical programming practices, including controls over hard-coding (Module 5)
□ Version control of statistical reporting and output (Module 5)
□ Requirement for an audit trail to link output used in a report or publication back to programming output (Module 5)
□ Validation of statistical programming and quality control checks of the statistical analysis process (Module 5)
□ Computer system validation (Module 5)
□ Specification, production and control of the randomisation schedule/code (Module 3)
**Areas usually requiring statistical input/involvement**
□ Statistical input into trial design and protocol development, including sample size validation (Module 3)
□ Maintenance of blinding and procedures for unblinding for analysis (Module 3)
□ Development and review of case report forms (CRFs) (Module 4)
□ Review of database specification and data validation plan (Module 4)
□ Central/statistical monitoring (Module 4)
□ SAE reconciliation (Module 4)
□ Use and validation of non-CRF data (e.g. central laboratory data) (Module 4)
□ Coding free text fields (Module 4)
□ Data lock and processes for obtaining the data for analysis (Module 4)
**General GCP principles which extend to statistical processes**
□ Quality systems, written procedures, etc. (Module 2)
□ Training documentation (Module 2)
□ Trial master files and archiving (Module 2)
**Core GCP material**
□ Introduction to GCP (Module 1)
□ UK regulations, frameworks and guidance and ICH GCP (Module 1)
□ Principles of GCP (Module 1)
□ Roles and responsibilities (Module 1)
□ Informed consent (Module 1)
□ Safety reporting definitions (Module 1)
□ Serious breaches (Module 1)

Feedback from the UKCRC CTU Statistics group activities indicated a desire for training to be stand-alone, which resulted in the additional generic module on core GCP principles (Module 1) to be developed. The modular approach follows the logical order of the progressive stages of a clinical trial, from trial design through to data analysis and reporting. The final module (Module 5) contains content most relevant to statistical programming and analysis incorporating recommendations from other UKCRC statistical practice guidance. References are provided to point to more in-depth guidance.

The modular format allows flexibility regarding delivery (face to face or e-learning) either as stand-alone training or to supplement usual local GCP training practices. Face-to-face training is intended for delivery within statistics teams where the lead training provider is an experienced researcher with a good understanding of local processes. Group activities in a face-to-face small group teaching environment provide an opportunity to consider how GCP principles can be implemented in line with local statistical practice and standard operating procedures, including consideration of risk proportionate approaches given the variability in trials’ portfolios across CTUs. Translation of the training material to e-learning is crucial for accessibility. E-learning is essential for statisticians working in research teams but isolated from other statisticians, allows immediate access for new statisticians and allows accessible continued professional development. Training certificates document specific modules completed.

### Critical review and piloting

During the development phase, updates were provided, and feedback was received at 6-monthly meetings of the UKCRC CTU Statistics Group. This feedback provided direction to both the content and format for presentation. A draft version of the complete training package was piloted through small group face to face training in five CTUs (Oxford, Royal Marsden and Imperial Cancer Research, Edinburgh, Leeds, Newcastle) and took approximately 2 h to deliver, up to 3 h including exercises and discussion. A senior statistician within each CTU delivered draft versions of the slide content and provided collective feedback regarding understanding, relevance and practical implementation. It received overwhelmingly positive feedback: “this is a really valuable tool to add to our training”; “something the stats community definitely needs and pleased that this is being taken forward”; “all-in-all that was a very positive experience”; “the general feeling was that it was a lot more useful than an afternoon spent at standard GCP training”; “people were engaged… thinking if any [local] practices could be improved.” The face-to-face engagement was particularly highlighted: “the face-to-face aspects are particularly useful as this enabled us to discuss the various aspects in relation to our CTU SOPs, processes and documentation etc.” as was the relevance to new starters: “I wish I’d had this when I first started out.” The feedback was extensive and detailed including suggested amendments to content, presentation and language in order to clarify ambiguities. Following feedback, the training materials were extended to incorporate (i) training certificates, (ii) core regulatory GCP principles to save having to complete two courses and (iii) a recommendation for frequency every 2 years.

Additionally, a standalone, day-long, small group teaching session, based on updated draft versions of the slide content and accompanied detailed notes, was delivered by members of the development team, to NIHR staff in an external unit working on non-clinical trial NIHR funded research, including statisticians and data management staff. Fourteen of the attendees provided formal written feedback (Table 3): all would recommend the session to a fellow researcher, four scored the session 5/5 (excellent), the remainder scoring 4/5; 13 respondents “strongly agreed” or “agreed” the training material was relevant to their role, and all “strongly agreed” or “agreed” that the training increased their learning and understanding of GCP requirements in relation to their role. Useful, supportive comments included “far more relevant to ‘real life’ than basic GCP training,” “essential info for trials researchers,” “informative, relevant, good structure,” “comprehensive” and “this course should be made available to anyone using/collecting data rather than just statisticians. All of the people who work with statisticians should work as a team and therefore be offered similar training opportunities where roles/activities overlap.”

**Table 3:** Quantitative feedback from pilot work

	NIHR Statistics Group (*N* = 11 groups)		NIHR Unit (*N* = 14 staff)	
The training material was relevant to my role				
Strongly agree	5	45%	8	57%
Agree	6	55%	5	36%
Neutral	0	0%	1	7%
The training material increased learning and understanding of GCP requirements in relation to my role				
Strongly agree	6	55%	7	50%
Agree	3	27%	7	50%
Neutral	1	9%	0	0%
Not answered	1	9%	0	0%
I will be able to apply new learning/skills				
Strongly agree	5	45%	3	21%
Agree	5	45%	10	71%
Neutral	1	9%	1	7%
The session was clear and well presented				
Strongly agree	5	45%	8	57%
Agree	5	45%	6	43%
Neutral	1	9%	0	0%
The session was interesting and relevant				
Strongly agree	4	36%	6	43%
Agree	6	55%	7	50%
Neutral	1	9%	1	7%

The training was summarised at an invited parallel workshop at the NIHR Statistics Group annual meeting to ensure applicability outside of the UKCRC Registered CTU network. Structured, anonymous feedback from eleven roundtable groups (Table 3) demonstrated that every group either “strongly agreed” or “agreed” that the training material was relevant to their role. The majority (82%) “strongly agreed” or “agreed” that the training increased their learning and understanding of GCP requirements in relation to their role. A final draft was circulated to the MHRA for comment.

### Dissemination

The Good Statistical Practice training materials are freely available and accessible through a variety of portals:

1. To CTUs via the UKCRC online platform https://www.ukcrc-ctu.org.uk/
2. To NIHR researchers via the NIHR Learn platform https://learn.nihr.ac.uk/
3. Worldwide via the University of Leeds Institute of Clinical Trials Research https://medicinehealth.leeds.ac.uk/leeds-institute-clinical-trials-research

The material lends itself to statistical team training, pre-conference training at relevant statistical and/or clinical trials conferences as well as individual learning.

An oral presentation at the International Clinical Trials Methodology Conference [18] shared the need, development and pilot work and outlined content and modules, initiating wider dissemination activities and global awareness. The Good Statistical Practice training was launched in an accessible UKCRC led Trial Methodology Research Partnership webinar [19].

As a UKCRC CTU initiated project, the network will be able to update the material as necessary, monitor access metrics and measure longer-term impact.

## Discussion

Statisticians are fundamental in ensuring clinical research, including clinical trials, are conducted with quality, transparency, reproducibility and integrity. All research staff should have training in GCP and an awareness of GCP requirements in relation to their role [4–6] and an understanding of translation into practice GCP training undertaken by most academic statisticians is generic intended for site delivery staff. Crucially, it does not cover activities related to their role, resulting in variation in awareness, interpretation and practical implementation of relevant regulatory requirements and recommendations for good practice with regards to statistical conduct.

The Good Statistical Practice (GCP for Statisticians) project has been developed and extensively piloted new, role-specific, comprehensive, accessible GCP training material tailored to statisticians working in clinical research, particularly the clinical trials arena. The training equips statisticians with relevant regulatory knowledge and strengthens GCP interpretation and implementation in relation to statistician responsibilities to encourage best practice, leading to transparent and reproducible statistical activity as required by regulatory authorities. The Good Statistical Practice (GCP for Statisticians) training material is directly relevant to all statisticians working in the medical arena and is freely available for national and international adoption.

## Limitations

The development and piloting of the Good Statistical Practice (GCP for Statisticians) material was conducted by the UKCRC Registered CTU Statisticians Operational Group and the NIHR Statistics Group. As such, the training materials are based on UK Medicines and Healthcare products Regulatory Agency (MHRA) Good Clinical Practice guidance and tailored to UK statisticians. The training materials are intended to enable discussions to encourage best statistical practice tailored against local standard operating procedures and hence can be used worldwide as an evaluation of local processes for translation into good statistical practice as required by local regulatory authorities.

## Conclusions

The Good Statistical Practice (GCP for Statisticians) project has developed and extensively piloted new, role-specific, comprehensive, accessible GCP training tailored to statisticians working in clinical research. This role-specific training will encourage best statistical practice, leading to transparent and reproducible statistical activity, as required by regulatory authorities and funders.

## Data Availability

All survey data generated are included in this published article. Data sharing is not applicable as no datasets were generated.

## Abbreviations

CTIMP: Clinical Trials of Investigational Medicinal Products
CTU: Clinical Trials Unit
EMA: European Medicines Agency
GCP: Good Clinical Practice
GSP: Good Statistical Practice
HRA: Health Research Authority
ICH: International Council for Harmonisation of Technical Requirements for Pharmaceuticals for Human Use
MHRA: Medicines and Healthcare products Regulatory Agency
MRC: Medical Research Council
NIHR: National Institute for Health and Care Research
TMRP: Trial Methodology Research Partnership
UKCRC: UK Clinical Research Collaboration
